# Study protocol for a randomised controlled trial of haloperidol plus promethazine plus chlorpromazine versus haloperidol plus promethazine for rapid tranquilisation for agitated psychiatric patients in the emergency setting (TREC-Lebanon)

**DOI:** 10.12688/f1000research.19933.1

**Published:** 2019-08-15

**Authors:** Joseph E. Dib, Clive E. Adams, Werner Henry Ikdais, Elie Atallah, Hiba Edward Yaacoub, Tony Jean Merheb, Francois Kazour, Fouad Tahan, Georges Haddad, Marouan Zoghbi, Jocelyn Azar, Chadia Haddad, Souheil Hallit

**Affiliations:** 1 Institute of Mental Health, University of Nottingham, Nottingham, Nottinghamshire, NG1 1NU, UK; 2 Institution of Mental Health, University of Nottingham, Nottingham, Nottinghamshire, UK; 3 Psychiatric Hospital of the Cross, Deir Salib, Jal l Dib, Lebanon; 4 Faculty of Medicine and Medical Sciences, Holy Spirit University of Kaslik, Beirut, Lebanon; 5 Faculty of Sciences, Lebanese University of Beirut, Beirut, Lebanon; 6 School of Medicine, Lebanese American University, Beirut, Lebanon; 7 Department of Psychology, Holy Spirit University of Kaslik, Beirut, Lebanon; 8 Faculty of Medicine, St Joseph's University, Beirut, Lebanon; 9 INSERM U930, équipe 4 “Troubles affectifs”, Université François-Rabelais de Tours, Tours, France; 10 INSPECT-LB: Institut National de Sante Publique, Epidemiologie Clinique et Toxicologie, Beirut, Lebanon

**Keywords:** Lebanon, Randomised Controlled Trial, Rapid Tranquilisation, Chlorpromazine, Haloperidol, Promethazine, Agitation, Aggression, Violence, Trial Protocol, Emergency management

## Abstract

**Background: **Agitated and aggressive behaviours are common in the psychiatric setting and rapid tranquilisation is sometimes unavoidable. A survey of Lebanese practice has shown that an intramuscular haloperidol, promethazine and chlorpromazine combination is a preferred form of treatment but there are no randomised trials of this triple therapy.

**Methods:** This is a pragmatic randomised trial. Setting - the psychiatric wards of the Psychiatric Hospital of the Cross, Jal Eddib, Lebanon. Participants - any adult patient in the hospital who displays an aggressive episode for whom rapid tranquilisation is unavoidable, who has not been randomised before, for whom there are no known contraindications. Randomisation – stratified (by ward) randomisation and concealed in closed opaque envelope by independent parties. Procedure – if the clinical situation arises requiring rapid tranquilisation, medical residents overseeing the patient will open a TREC-Lebanon envelope in which will be notification of which group of treatments should be preferred [Haloperidol + Promethazine + Chlorpromazine (HPC) or Haloperidol + Promethazine (HP)], along with forms for primary, secondary and serious adverse effects. Treatment is not given blindly. Outcome - primary outcome is calm or tranquil at 20 minutes post intervention. Secondary outcomes are calm/tranquil at 40, 60 and 120 minutes post intervention, asleep, adverse effects, use of straitjacket and leaving the ward. Follow-up will be up to two weeks post randomisation.

**Discussion: **Findings from this study will compare the HPC versus HP combination used in Lebanon’s psychiatry emergency routine practice.

**Trial registration: **ClinicalTrials.gov NCT03639558. Registration date, August 21, 2018.

## Abbreviations

HPC: Haloperidol + Promethazine + Chlorpromazine; HP: Haloperidol + Promethazine; TREC: Abbreviation for the Portuguese ‘Tranquilização Rápida-Ensaio Clínico’ or Rapid Tranquilisation Clinical Trial; RT: Rapid Tranquilisation; ER: Emergency Room; DMC: Data Monitoring Committee; SC: Steering Committee

## Introduction

Aggressive and violent behaviour is a common behaviour seen in emergency psychiatric presentations with a prevalence of 3–10% ^1^. This aggression is due to a range of psychiatric disorders, such as schizophrenia and bipolar disorder, and/or substance use, personality disorders or dementia ^2^. Guidelines recommend aggressive patients to be ‘verbally tranquilised’ or some form of de-escalation in order for the attending physician to accurately and safely perform a diagnostic history and physical examination ^3^. Aggressive patients make this process difficult or sometimes impossible and carers may be required to work with limited evidence. Since the psychiatric team has a responsibility of ensuring the safety of everyone, rapid and safe tranquilisation may become unavoidable.

### Rapid tranquillisation

Rapid tranquillisation (RT) is not a ‘treatment’ but rather a short-term management technique for severely agitated and/or aggressive behaviour in people experiencing severe psychiatric distress. Due to its restrictive nature, RT is a last resort when all other attempts to calm a situation have failed and should always be used in a way that respects human rights and never as a substitute for adequate staffing ^4^.

In this difficult situation medication(s) are most commonly administered intramuscularly ^5^, and, depending on where in the world the management is happening, physical restraints may include use of a straitjacket ^6^, seclusion room ^7^ or medical restraints ^8^ - binding the patient safely to a bed using two or four points ^9^. Physical restraining by staff to administer medication is common worldwide.

### Guidelines

Globally, guidelines differ in their specific recommendations ^4, 10, 11^ – often based on the same limited evidence - and then may not be adhered to in local clinical practice ^12, 13^. There are no directly relevant national Lebanese guidelines.

### Local practice

In preparation for this study we surveyed RT practice in the largest (>800 beds) psychiatric hospital in Lebanon (Psychiatric Hospital of the Cross, Beirut). Several different medications were used but the use of a combination of haloperidol plus promethazine plus chlorpromazine (HPC) was common ^13^. Haloperidol plus promethazine (HP) – without the addition of chlorpromazine - was also used. Long clinical experience has proved both combinations effective, but which is best in terms of overall safety remains unclear.

### Existing evidence

Our systematic searches found that the HPC combination is used elsewhere ^14^ but identified no relevant randomised trials. The HP combination, however, has strong trial-based evidence from low and middle-income countries supporting its use - albeit in comparison with medications other than HPC ^15^. There is an unanswered question as regards the relative effects of HPC versus HP.

### TREC-Lebanon

This study takes its name from the first Brazilian TREC study (TREC – abbreviation for the Portuguese ‘Tranquilização Rápida-Ensaio Clínico’ or Rapid Tranquilisation Clinical Trial) ^16, 17^ indicating the debt we owe to their pragmatic design and to allow easy identification with an increasing group of related randomised trials.

### Objective

TREC-Lebanon aims to compare its routine emergency drug HPC versus HP during an agitated episode requiring rapid tranquilisation.

### SPIRIT checklist

The SPIRIT 2013 statement which consists of a 33 item checklist of minimum recommended items ^18^. More information can be found on the SPIRIT website ( Table 1).

**Table 1.** SPIRIT Checklist (TREC-Lebanon).

	STUDY PERIOD						
	Enrolment	Allocation	Post-allocation				Close-out
TIMEPOINT**	*-t _1_*	0	*t _1_*	*t _2_*	*t _3_*	*t _4_*	*t _x_*
**ENROLMENT:**	X		20 MINUTES	40 MINUTES	60 MINUTES	120 MINUTES	2 weeks
**Eligibility screen**	X						
**Informed consent**	X						
***[List other procedures]***	X						
**Allocation**		X					
***[Intervention A]***	Haloperidol + Promethazine	X	X	X	X	X	
***[Intervention B]***	Haloperidol + Promethazine + Chlorpromazine	X	X	X	X	X	
**ASSESSMENTS:**							
***Aggression***	X	X	X	X	X	X	
***Tranquillity***			X	X	X	X	X
*** Demographics***	X						X
*** Adverse effects***			X	X	X	X	
*** Sleep***			X	X	X	X	
***Straitjacket***			X	X	X	X	
***Left the ward***	X	X	X	X	X	X	X

### Setting

Lebanon is a low to middle income country in the Middle-East with a population of approximately 6 million ^19^. The country has three mental hospitals and five psychiatric units within general hospitals (43 psychiatric beds per 10,000; 1 psychiatrist per 100,000) ^20^. The Lebanese Ministry of Health contracts the private sector to provide free treatment for patients who cannot afford to pay (the majority) ^20^. There are no disability benefits for people with mental disorders and no disability funding for mental health ^21^. Psychiatric Hospital of the Cross, set in the metropolitan area of Beirut (>2 million people), provides a service that extends across the whole country.

## Methods

TREC-Lebanon is a pragmatic trial designed by the main researcher (JD) working in close collaboration with partners in the Psychiatric Hospital of the Cross, Jal Eddib, Lebanon. 

This is the final version of the protocol.

### Eligibility

***Inclusion criteria.*** An adult (18–64 years) will be eligible if, when presenting at the Psychiatric Hospital of the Cross:

i. he/she requires emergency acute intramuscular medication because of disturbed and dangerous behaviour thought due to psychiatric morbidity; and
ii. if the clinician is uncertain of the benefits of the HPC combination over those of HP used together.

***Exclusion criteria.*** A person will not be eligible if:

i. The clinician
  • knows one treatment regimen has benefit over the other for that particular person;
  • is aware of a contra-indication of one of the treatments, such as
    ■ allergy;
    ■ past adverse reaction; or
    ■ already given/taken drugs in the community which would make additional HP or HPC ill-advised;
  • does not want to enter the person into the trial for any reason
ii. There is an Advanced Directive expressing a wish for one or other, or another treatment in the emergency setting;
iii. The person has already been randomised into the trial; and if
iv. An accompanying person (friend/family/Police Officer) refuses patient trial entry.

### Interventions

Haloperidol, promethazine and chlorpromazine are included in the WHO's List of Essential Drugs ^22^.

***Chlorpromazine.*** Chlorpromazine is a widely used ^23^, effective ^24^ antipsychotic drug, but can cause a number of adverse effects including anticholinergic and antihistaminic effects ^25^. Chlorpromazine is known to be the most epileptogenic of the older antipsychotic drugs causing seizures ranging from 1–4% depending on dosages ^26^.

***Haloperidol.*** Haloperidol is also an older effective ^27^ antipsychotic, less prone than chlorpromazine to cause sedation (less antihistaminic effects) but more causative of movement disorders including acute dystonia (involuntary dramatic contractions of muscles in, for example, the neck, face, pelvis, spinal muscles) ^28^. These acute reactions are not life-threatening but are distressing and frightening to the patient, further eroding trust in the services. Acute dystonia can be swiftly and successfully treated with drugs with anticholinergic/antihistaminic properties, such as promethazine or procyclidine ^29, 30^. The occurrence of these reactions, in 1–2% of those given haloperidol alone, was the cause of the early termination of the second TREC trial of Brazil (comparing HP with haloperidol alone) ^31^. The Steering Group of that study felt the evidence was strong enough to make the sole emergency use of intramuscular haloperidol impossible to justify if promethazine was available.

***Promethazine.*** Promethazine hydrochloride is also an old antipsychotic drug but also has potent anticholinergic and antihistaminic properties. This helps offset sickness and movement disorders – including acute dystonia – but also causes it to be sedative ^32^.

***The combinations.*** Combining drugs can change - increase or decrease - the incidence of known adverse effects ^33^ or result in novel effects unheard of with each drug on its own ^34, 35^. The HP combination is well tested, widely used and trusted. Evidence-based guidelines now recommend its use ^36^. We think the triple combination of haloperidol, promethazine and chlorpromazine (HPC) is not so widely employed, but do know it is not only Lebanon that uses it 17. Whether addition of another drug (chlorpromazine) has benefit or causes difficulties may be illustrated by this study, but we identified no existing literature suggesting that there are particular concerns regarding adverse effects of this HPC combination.

### Sample size and statistical considerations

The primary aim of TREC-Lebanon is to investigate whether the proportion of patients calm/tranquil at 20 minutes is any different between the two investigative approaches. In such a stressful situation, a small advantage for an intervention could represent a worthwhile benefit. However, to highlight a clear difference with confidence, this would need larger numbers of people than this phase of work would allow ( Table 2). Realistically, taking into account our preliminary work ^13^ and the time constraints of this PhD project with no extramural funding, TREC-Lebanon expects to involve a minimum of 90 patients across a 3-month period. Confidence intervals will be calculated and interpreted according to Altman ^37^. Statistical significance at the 5% level for the primary outcome and at 1% for the secondary outcomes. *K* statistics will be used for estimating inter-rater agreement for the primary outcome. SPSS version 24 will be used for the analysis of statistical data. 

**Table 2.** Size and statistical considerations.

Haloperidol + Promethazine (% tranquilised)	Total N required by % difference			
	10%	15 %	20%	25%
5	280	150	**98**	**70**
10			**124**	**86**
15				**98**
20				**108**
25				**116**

### Randomisation

Randomisation will be undertaken in the United Kingdom (by CEA). Small block sizes were chosen to ensure even distribution of treatments (4,6) and whether HPC was to be coded as '1' or '0' was randomly assigned using MS Excel's RAND function (HPC coded as '1'). A free online programme randomised block size, and then treatments to groups within blocks. Using our survey data ^13^, it was thought that 30% of randomised incidents would be on the women's ward - so the first set of complete blocks covering this proportion (therefore up to #32) were taken for the women's ward and men were then started at number 33. Tables of TREC-Envelopes’ number by contents will be constructed and supplied to a Lebanese colleague (SH). The tables will list the contents of the envelopes in groups of ten, not disclosing the block sizes used. SH, always working independently of both the TREC-Lebanon team and CEA, will ensure that the correct medication combination is named within each TREC-envelope before it is sealed.

Concealment of allocation will be ensured by i) not disclosing the randomly varied block sizes to the colleagues packing the envelopes; ii) the supply of tables to those colleagues that gives no suggestion that blocks are even being employed; iii) the independence of those packing the envelopes from the other researchers or the clinicians; and iv) the identical nature of the packed fully opaque envelopes.

These easy-to-use envelopes will be constructed of cardboard, identical and consecutively numbered. The final check to ensure that nothing has gone wrong with the randomisation will be by the Principal Investigator (CH) filling in a form for each block of ten opened envelopes. At analysis the date and time of instigation of treatment will be ordered and this will order the TREC-IDs showing that each envelope was opened consecutively.

### TREC-Lebanon is blinded for the initial ratings only

Because the TREC-Lebanon study evaluates care in the emergency situation, it is imperative that the doctors and nurses know which intervention is being given. The study is blind only up until the time that the TREC-Lebanon trial envelope is opened. Therefore, it is crucial that the evaluation of the severity of a person's disturbance and the first impression on the possible cause for the disturbed behaviour are recorded *before* the envelope is opened. Once the envelope is opened, doctors and nurses will have knowledge of the medications to be used. It is perfectly feasible that the knowledge that one drug has been given will influence the care beyond the actual effects of the medication. Keeping the study open from that point onwards is not only practical in the emergency situation, but also desirable as the evaluation of care being undertaken is as near real-world circumstances as is possible.

### Protocol registration

This trial has been registered and accepted at clinicaltrials.gov: https://clinicaltrials.gov/ct2/show/NCT03639558 (registered prospectively on August 21, 2018).

## Procedures

### Pragmatism

All trial materials, and guidelines for their use, are provided in the TREC-Lebanon folder supplied by the co-ordinating centre. The TREC-Lebanon trial is designed to not interfere with routine care. The eligibility criteria are simple and the process of randomisation fits closely into normal hospital procedures. Data collection will be limited to the minimum necessary and will involve little more than extraction of routine information by a person designated to spend time on the TREC-Lebanon trial. No data are redundant. It is not envisaged that busy doctors and nurses will spend time filling out complicated forms.

Blinding raters would have added additional complexity to the study that would have made the trial much less acceptable to the emergency room (ER) staff. More importantly, it would have completely changed the emphasis of TREC-Lebanon. What is being evaluated is the real-world practice of giving two different drug regimens in the psychiatric emergency setting. In the real-world situation health care professionals know what treatment is being given.

### In the community

Occasionally patients are given sedative medication by their parents, friends or law officials prior to psychiatric admission ^13^. As far as the hospital clinician is concerned, he or she still decides if the patient should be randomised within the trial if the patient is still exhibiting an agitated episode.

### Arrival at the hospital

Most people arrive at the hospital’s administrative centre whereby they are registered and then transferred to their ward ^13^. If patients are presenting with violent conduct that could potentially harm people in their vicinity, they are taken directly to their ward while those who brought them in fill out their paperwork – including all necessary consent forms (see section below).

### Triage to randomisation

Unlike TREC-Rio ^17^ whereby agitated patients arriving at the psychiatry ER have been given an intervention after the nurse opens the box within the ER, TREC-Lebanon operates differently due to the hospital not having an ER, prompting a different approach. The TREC-Envelopes are placed in the underground research clinic accessible only to researchers and doctors. The envelopes are separated between men and women (stratified randomisation). Residents who are on duty will be granted access to the research director’s clinic where the envelopes are securely kept and will be given two envelopes in a consecutive manner – one for a man and one for a woman while the research assistant keeps track and notes which envelopes have been taken. The resident carries the envelope during duty and in the event of an agitated episode, opens the envelope depending on sex, fills out the form, returns all forms within the envelope and returns it back to the research director’s clinic whereby completed forms will be stored in a separated secured TREC-box.

Whenever possible, carers accompanying the patient should have an opportunity to see the information leaflet ( *Extended data:* TREC-Lebanon information for relatives) before randomisation.

If the attending doctor decides the person should not be entered into TREC-Lebanon, he/she will notify the resident on duty and anonymised information on this group will be collected:

- date and time
- age
- gender
- ethnicity (if applicable)
- the reason not eligible for trial participation, or if they are eligible but failed to be randomised.

**Figure 1.**
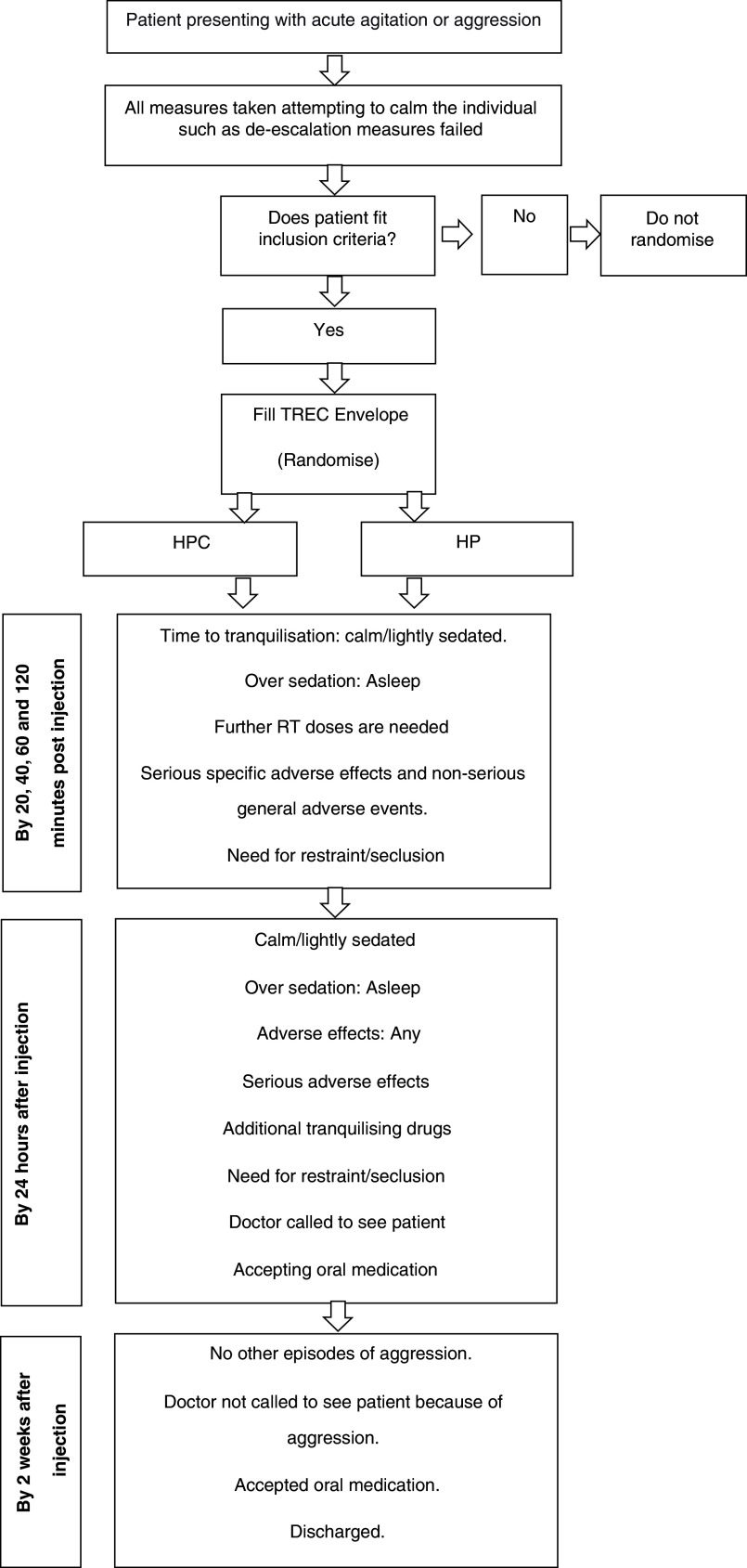
Flow diagram.

If the attending clinician decides the pateint to be eligible, then the next consecutive envelope is taken from the research director’s clinic, the form on its cover is filled out, and only then is it opened. The trial entry form printed on the sealed envelope ( *Extended data*: TREC-Envelope entry form) records brief baseline details about the person, the severity of disturbance, its presumed cause, the date and time of opening and the name of the doctor. This action constitutes trial entry ( Figure 1).

### Residents

The residents for the trial are WHI, HEY, EA and TJM. The hospital undergoes frequent resident rotation; therefore, residents undergoing training within the hospital for long durations of time were recruited in order to begin the trial, train incoming residents and see the trial completed. All residents, the Research Director (SH) and the Principle Investigator (CH) are involved in a WhatsApp group chat to inform, keep track and ask questions should they arise during the course of the trial.

### Trial envelopes

Every envelope has the entry form printed on it. Randomisation proceeds using a local pack system with the trial team providing the identical sealed envelopes made of sturdy fully opaque card.

Each envelope contains:

- Paper slip indicating the use of Haloperidol + Promethazine (HP)
- 1 × TREC-Lebanon follow-up form ( *Extended data:* Primary measures of outcome form)
- 2 × TREC-Lebanon stickers for the drug prescription form and medical notes

Or

- Paper slip indicating the use of Haloperidol + Promethazine + Chlorpromazine (HPC)
- 1 × TREC-Lebanon follow-up forms ( *Extended data:* Primary measures of outcome form, Dr. Stopwatch form and TREC-Lebanon main data collection form)
- 2 × TREC-Lebanon stickers for the drug prescription form and medical notes

*All doses used are at the discretion of the attending clinician*. If the contents of a trial envelope are destroyed, or unfit for use, the person should *not* be randomised a second time and the equivalent material should be obtained from the usual hospital supplies.

In the event of continuing aggression despite the TREC-Lebanon medication, ongoing emergency management is entirely at the discretion of the clinicians; another envelope *is not* opened.

### Outcome and follow-up

It is crucial that follow-up is complete and accurate for everyone entered into the study. As a pragmatic study, causing minimal interference with routine care, TREC will not employ any rating scale outcomes. It is likely that completion of scales would be inaccurate and incomplete, validity and reliability would be in question and clinical utility problematic. The main outcome of TREC-Lebanon is tranquillisation by 20 minutes. This was the primary outcome requested by the nursing and medical staff of the hospital ^13^. By asking the relevant clinical staff to select the primary outcome for TREC-Lebanon we hoped to ensure maximum compliance with the trial protocol. Therefore, upon injection of the patient, a timer is started on the resident’s phone and this rings at 20 minutes and then again at 40, 60 and 120 minutes. At each period the attending resident rates whether the person, in their opinion, is tranquil, asleep, has shown adverse effects or needs additional treatment and records this on the follow-up form found originally within the sealed envelope ( *Extended data:* Primary measures of outcome form). The person is considered tranquillised when they are felt to be calm and peaceful, but not asleep. They should not be agitated or restless, nor displaying threatening verbal behaviour, physical aggression against objects, self-aggression or physical aggression to other people.

The Dr. Stopwatch form ( *Extended data*) is included within the envelope and contains two boxes: time the resident felt the patient was tranquilised either tranquil or asleep and a comments box. The tranquilised box is the ‘true tranquilisation’ outside the 20, 40, 60 and 2 hour time intervals within the primary measure of outcome form, assuming the patient becomes tranquilised after the 2 hour maximum limit. The additional comments box is included if the resident needs to note additional information (i.e. why the patient did not calm down within the 2 hour time frame). The resident starts their own personal stopwatch from the time of intervention to when he/she feels the patient has become calm/tranquil or asleep.

Additional data are recorded at 24 hours and finally at two weeks ( *Extended data:* TREC-Lebanon main data collection form). These additional data are to be extracted from routine notes. If the patient is transferred to another hospital, the co-ordinating centre will contact every relevant hospital to find out further details on what happened after transfer.

### Data collection, entry and analysis

All data for TREC-Lebanon will be transcribed and collated from the TREC-envelope forms, the follow-up form, severe adverse event form and routine notes of each ER or ward into a form within MS Access – a database management system from Microsoft ( *Extended data:* TREC-Lebanon main data collection form). These anonymised data, in compliance with the ethics committee requests, will be protected as the hard copy forms do not leave the research director’s clinic. All transcribed raw data used by the main researcher (JD) will have personal information such as names of patients abbreviated to retain anonymity.

Analysis will take place using Statistical Package for Social Sciences (SPSS) ^38^. This will take the form of simple frequencies – to test the integrity of the data, and, for binary outcomes, relative risks and respective 95% confidence intervals, and for continuous data mean differences and their 95% confidence intervals. Tables for this analysis are prepared *before recruitment of the first patient* ( *Extended data*: Dummy statistical tables). No additional analyses are anticipated. All analysis will be based on groups as randomly allocated; this will be an intention-to-treat analysis.

All data that includes private information will be anonymised. Once the study is completed, access to raw data containing personal patient information will only be accessible to the hospitals’ director and responsible clinician.

## Anticipated risks

### Ineligible people entering the study

It is possible though highly improbable that patients who do not fit the trial’s entry criteria may enter the study. Those that do will not be counted as part of the trial and their notes will be disregarded. Detecting ineligible patients will be seen in the data entry form left in a bin on each ward (the TREC-bin into which all filled out envelopes are put) making it simple and direct to trace. 

### Staff compliance with protocol

Attending resident and nurse should monitor action of given intervention, i.e. make sure given treatment has been injected properly. In the event that an agitated patient may break any of the treatment tools – mainly the syringe containing the treatment intervention or destroy the vial containing the intervention before being placed within the syringe, another TREC envelope should not be opened. Instead, attending resident should carry on as per hospital protocol and fill out the serious event form detailing the circumstances ( *Extended data:* Serious event form). Nurses should also detail the nature of compliance as they would normally do in their notes. In the event a patient does break the syringe or capsule, the situation is rectified with the nurse bringing the emergency treatment as detailed in the paper slip in the envelope.

### Feasibility phase

A feasibility phase will take place before the trial commences. The feasibility phase will include a limited number of envelopes (i.e. 5) with contents known to the trialists. The feasibility phase is designed to test the trial’s procedure in practice in order to assess if any unforeseen circumstances arise. In the event such unforeseen circumstances do arise, the trialists will rectify and mediate in the most practical way possible. Changes will be noted by the main researcher (JD) and updated in the trial protocol.

### Toxicity and serious unexpected events

After trial entry, clinical events are recorded, as usual, in the patients' notes. Complications and adverse events should be managed as usual. A serious unexpected event form ( *Extended data:* Serious event form) is provided and will be sent to the TREC-Lebanon Co-ordinator (JD) as soon as it is completed.

## Ethics approval and consent to participate

The Helsinki Declaration ^39^, the European Directive on Clinical Trials ^40^ and the Nuffield Council documents on bioethics ^41^ state that trials in non-consenting patients are permitted on two conditions: i) no other context exists in which to answer the question; and ii) all trial participants receive clear therapeutic benefit from whichever arm they are randomised to. Consideration also has to be given to the local legislation ^21^, namely:

1. Lebanese Act no. 72-9/9/1984 Welfare Act and Protection and Treatment of Mentally Ill Patients;
2. Lebanese Act no. 673-16/3/1998 Narcotic Drugs and Psychotropic Substances and Precursors;
3. Lebanese Act no. 220-29/5/2000 Rights of Mentally Handicapped in Lebanon; and
4. Lebanese Act no 574-11/2/2004 Patients’ Rights and Informed Consent;

which also protects the rights of patients and their families and carers.

Aggressive patients in a situation of psychiatric emergency are not able to give consent for their participation in a study ^42^ as their aggressive state, along with their psychiatric disorder places them in a state whereby they lack the capacity to do so, e.g. a patient with schizophrenia suffering from hallucinations is in a state where he or she is not mentally competent to understand the conditions of the treatments involved ^43^. In the case where parents or legal guardians are unavailable, a third party must confirm to the best interest standard whereby the decision made is the most beneficial to the patient. This is a direct application of the principle of beneficence and proportionality: maximize benefit and avoidance of harm. The substituted judgment standard aims to implement the subjective preferences of the patient ^44^.

In routine care medication is usually given against the will of the patient. Therefore, for TREC-Lebanon, in the same way that doctors are responsible for the choice of a treatment in routine care, they take responsibility for the recruitment of a patient into the study. TREC-Lebanon will not involve administering an inactive compound to those who clearly need sedation/tranquillisation. Both treatments calm aggressive disturbed people ^13^, so there is no 'experimental' intervention. What is still uncertain is the speed for the onset of action, the duration of the effects and the different kinds of adverse reactions. TREC-Lebanon will attempt to answer clinical questions to help the care of this group of people.

A patient/carer information leaflet about TREC-Lebanon is available for all for whom a TREC-Lebanon envelope is opened (in English and Lebanese Arabic). Carers will always be free to decide that their relative should not be entered. Not being involved in TREC-Lebanon will not affect the person's standard of care. An information sheet is provided detailing the aim and purpose of the study ( *Extended data:* TREC-Lebanon information for relatives).

Patients who do not have parents or legal guardians will still be randomised if they are aggressive and fit the inclusion criteria, but will be given a patient consent form post randomisation detailing the background of the study and their right to withdraw all personal data from the trial for whatever reason they see fit ( *Extended data:* TREC-Lebanon consent form for patients)

Ethics Application File from the Hospital of the Cross was filled out by the trial coordinator detailing the background and procedure of the trial (non-consenting nature, data sharing agreement, liability waiver) were read and signed before gaining ethical approval and IRB letter.

TREC-Lebanon did not require ethics approval from the University of Nottingham (Ethics number: 271) and has passed ethics approval from the Psychiatric Hospital of the Cross, Beirut, Lebanon (Ethics number: HPC 001/2018).

### The Helsinki Declaration

The Declaration of Helsinki ^45^ requires the informed consent of participants in randomized controlled trials (paragraph 25). However, according to paragraph 28, if the individual for whatever reason is unable to provide consent, a legally authorised individual – usually a family member or guardian may provide on behalf. Finally, paragraph 30 states if no family members are present and the research is unable to be delayed, randomisation may proceed provided the specific reasons to why the patient is unable to provide consent (in this case, mental illness with violent display) and the research has been approved by an ethics committee. TREC-Lebanon abides by the Helsinki Declaration and provides a consent form to all patients who were randomised without having the ability to consent at the time or a family member/guardian to consent on their behalf.

## Trial Organisation

The TREC-Lebanon Co-ordinating Group: The co-ordinating centre of the Lebanese arm is based at the Institute of Mental Health, University of Nottingham, United Kingdom. The Co-ordinating Group has overall responsibility for the design of the proposed trial and is responsible for all aspects of day-to-day trial administration. The Co-ordinating Group is also responsible for preparing reports for the Steering Committee. Membership: JD, CEA, SH.

### Steering Committee

The overall progress of the trial, adherence to protocol, patient safety and the consideration of new information will be monitored by a scientific and administrative Steering Committee (SC). At the end of the proposed study period, the SC will consider the extension of the study, to allow the detection of other important effects. Membership: PS and RH.

### Data Monitoring Committee

TREC-Lebanon will include a committee to oversee progress of the trial. Since TREC-Lebanon might take three to six months to complete, an independent Data Monitoring Committee (DMC) will, in confidence, monitor results. This could be undertaken on a week to week or month to month basis depending on the collective agreement of all the members of the DMC. In the light of the interim data, and of any other evidence or advice they wish to seek, the DMC will inform the chair of the SC if, in their view: i) there is proof beyond reasonable doubt that for any particular group or subgroup treatment with one or other regiment is clearly indicated or contraindicated; or, ii) it is evident that no clear outcome will be obtained. Proof beyond reasonable doubt may be taken as the difference of at least three standard deviations and at least one of the primary outcomes.

The DMC may communicate certain interim analysis to the SC or suggest certain protocol changes, but the SC will remain responsible for deciding which changes to adopt. Membership: GA and JM. The committee will receive the first batch of data when trial participants are at a total of 50 along with information such as adverse effects, unforeseen circumstances and trial progress so far.

## Current Study Status

At the time of submission of this manuscript, trial was still ongoing albeit approaching its final phase. Presently, trial has concluded recruitment process. Data has been transcribed and awaiting analysis.

## Dissemination of Study Outcomes

The results of TREC-Lebanon will be published in at least one peer reviewed indexed journal and will be presented in relevant conferences.

## Discussion

As mentioned earlier, violence in the psychiatry setting is common and rapid tranquilisation is sometimes necessary and unavoidable. Not only are national guideline recommendations limited in their evidence backing - but surveys of practice have shown to differ from clinicians’ opinions on management during an agitated episode ^13^.

The HP and HPC combination have both been used in routine care outside Lebanese practice ^16^, but as far as systematic searching has shown, there exists no clinical trials randomising the HPC combination, making it an ideal candidate for randomisation.

Possible limitations for this trial are that there are no ERs; therefore residents must always carry the TREC envelopes at all times, increasing the risk for error (i.e. misplacing envelope, using wrong envelope, etc.). Despite the limitation, the chances of error remain low due to the small sample size of 100.

Overall, since TREC-Lebanon is comparing two interventions with drug combinations that are used in its routine practice ^13^, we assume in both cases that rapid tranquilisation will be achieved reducing the risk of the agitated patients harming themselves, as well as harming others.

## Data availability

### Underlying data

No underlying data is associated with this article.

### Extended data

Open Science Framework: TREC-Lebanon Protocol, https://doi.org/10.17605/OSF.IO/MYCQ9 ^46^.

This project contains the following extended data:

- TREC-Lebanon information for relatives
- TREC-Envelope entry form
- Primary measures of outcome form
- Dr. Stopwatch form
- TREC-Lebanon main data collection form
- Dummy statistical tables
- Serious event form
- TREC-Lebanon consent form for patients

Data are available under the terms of the Creative Commons Attribution 4.0 International license (CC-BY 4.0).
