# Supplementary material for: Study protocol for a randomised controlled trial of haloperidol plus promethazine plus chlorpromazine versus haloperidol plus promethazine for rapid tranquilisation for agitated psychiatric patients in the emergency setting (TREC-Lebanon)
Source: F1000Res. 2019 Aug 15;8:1442. [Version 1] doi: 10.12688/f1000research.19933.1 (PMC7262571; doi:10.12688/f1000research.19933.1)
Supplement: Peer review history

# Peer review: Reviewer response for version 1

**DOI:** 10.5256/f1000research.21877.r63478

**Published:** 2020-05-29

**Authors:** Gisele Huf

**Affiliations:** 1 National Institute of Quality Control in Health, Oswaldo Cruz Foundation, Rio de Janeiro, Brazil

This manuscript reports a protocol for a randomised clinical trial to evaluate the best pharmacological management of agitated patients in psychiatric emergency rooms in Lebanon. It follows a previous version of this protocol, published in 2002, for studies conducted in Brazil and India, assessing the use of the prevalent intervention in both countries, a combination of haloperidol and promethazine. The Lebanon study will compare this intervention with their local practice: a combination of haloperidol, promethazine and chlorpromazine.

 The similarities with the original protocol are many. The pragmatic design, the blinding up to the point of randomisation just to ensure concealment of allocation, the type of participants, the doses of medication at the discretion of the doctor, the main and secondary outcomes, and the analysis of data. The main differences are in the randomisation process: it is stratified by ward and delivered in sealed envelopes instead of sealed boxes containing the treatment.

 Although the trial intends to recruit only 90 participants, the results of this study could be added to the body of evidence in systematic reviews. I have only some minor comments:

1. The authors state they will perform an intention-to-treat analysis, but they also declare that if ineligible people enter the trial, they will not be counted and their notes will be disregarded. Isn’t it a contradiction?
2. The authors state that in the event that some syringe or vial containing medication is broken, another envelope should not be opened. It doesn´t make much sense.
3. The authors state that guidelines are “often based on the same limited evidence”. This is currently not accurate and the references used (1998, 2001 and 2004) are outdated.

Is the study design appropriate for the research question?

Yes

Is the rationale for, and objectives of, the study clearly described?

Yes

Are sufficient details of the methods provided to allow replication by others?

Yes

Are the datasets clearly presented in a useable and accessible format?

Yes

Reviewer Expertise:

Clinical epidemiology

I confirm that I have read this submission and believe that I have an appropriate level of expertise to confirm that it is of an acceptable scientific standard.

# Peer review: Reviewer response for version 1

**DOI:** 10.5256/f1000research.21877.r63552

**Published:** 2020-05-19

**Authors:** Stefan Leucht

**Affiliations:** 1 Department of Psychiatry and Psychotherapy, School of Medicine, Technical University of Munich (TUM), Munich, Germany

The authors submit the protocol of the clinical trial comparing to combinations of antipsychotics for emergency patients which need a rapid intervention.

 The protocol is a very detailed and explains every important step of the study. The team around the study, in particular the author Prof Clive Adams, has a lot of experience in this type of study. He has helped to contact and published several trials of this kind in the past.

 The current trial will fill another bit of the puzzle about the best intervention for acutely ill mentally ill patients who need rapid treatment. As the interventions are frequently used in the trial centre so that the clinicians have a lot of experience with them, I do also not see an important risk going beyond routine care.

Is the study design appropriate for the research question?

Yes

Is the rationale for, and objectives of, the study clearly described?

Yes

Are sufficient details of the methods provided to allow replication by others?

Yes

Are the datasets clearly presented in a useable and accessible format?

Not applicable

Reviewer Expertise:

Meta-analysis of treatments for schizophrenia, clinical trials.

I confirm that I have read this submission and believe that I have an appropriate level of expertise to confirm that it is of an acceptable scientific standard.

# Peer review: Reviewer response for version 1

**DOI:** 10.5256/f1000research.21877.r55715

**Published:** 2019-11-05

**Authors:** Sophie Hirsch

**Affiliations:** 1 Interdisciplinary Section of Neuro-Oncology, Department of Neurology, Hertie Institute for Clinical Brain Research, University Hospital Tübingen, Eberhard Karls University of Tübingen, Tübingen, Germany

This is a very important field of research, due to the fact that randomized controlled trials on people with severe mental illness showing aggressive behavior is still scarce and data for improving safety of this patient group is urgently needed. There are many positive points in the study design that should be highlighted: First, the opportunity for highly disturbed patients to give/withdraw informed consent for study participation after randomization to ensure that these patients are not excluded systematically and the organizational efforts that are made to keep the study recruiting even during night/weekend duty. Second, the blinded rating before the administration of the drug. Third, the 2 hours and 24 hours follow-ups which are missing in some of the rapid tranquilization studies guidelines have to rely on. And last but not least, the consideration of gender issues and the fact that patients who are not eligible for the study are also recorded.

 For this is a study protocol, it would have been better, to have it published before starting the recruitment process. Due to the fact, that the recruitment process is already completed comments or suggestions for improvement from the reviewers cannot be taken into account anymore. I would like to name some of these flags anyway: The evidence for the combination of haloperidole and promethazine is already quite good (evidence level 1a). Despite this high quality evidence, guidelines at least in Europe are constrained in recommending this combination due to safety reasons (especially QTc prolongation). At the moment, clinicians face the situation that a combination that is favored by international evidence and at least some clinical practice guidelines is not approved by the agencies. Adding a third active ingredient makes the adverse events and interdependencies even more unclear (e. g. chlorpromazine plus haloperidole and the risk for seizures). Against the background of this discussion and the recent discussion if the tranquilizing effect of a drug combination could also be achieved by a higher dose of one drug as monotherapy, the study design with a two- and a three-drug-combination and even the doses of these combinations left at the discretion of the clinician seems questionable. Especially, when rating the effects of the drugs at the follow-up points dosages and additional drugs should be analyzable per patient. It stays unclear if these longitudinal assessments are possible within the scope of the study. One sentence explaining the SPIRIT checklist would have been helpful.

 Concerning protection of data privacy, it would be interesting how the abbreviations for the patients were built up and which data was exchanged via the Whatsapp group.

 (Note to my "partly" answer on the last question: I missed the sheets that had to be filled in by the doctors. I did not understand why the form "Dr. Stopwatch" and "TREC-Lebanon main data collection form" only were present in the HPC- and not in the HP-envelope.)

Is the study design appropriate for the research question?

Yes

Is the rationale for, and objectives of, the study clearly described?

Yes

Are sufficient details of the methods provided to allow replication by others?

Yes

Are the datasets clearly presented in a useable and accessible format?

Partly

Reviewer Expertise:

Violence and coercion in psychiatry, targeted therapies in neurooncology

I confirm that I have read this submission and believe that I have an appropriate level of expertise to confirm that it is of an acceptable scientific standard, however I have significant reservations, as outlined above.

# Response

**Published:** 2020-05-19

**Authors:** Joseph Dib

**Affiliations:** University of Nottingham, UK

Hello,

On behalf of myself and the TREC-Lebanon team, we would like to thank you for taking the time and responding with valuable input pertaining to the protocol-manuscript.

To address your points: while it is true that it would have been better to have the protocol published before recruitment began, the authors (including myself) were on a limited time schedule and had to do with what time we were given. For instance, the resident doctors that were part of the trial team i.e. Administering the intervention, etc would have completed their rotation and left the hospital which would have compromised the study.

**Adding a third active ingredient makes the adverse events and interdependencies even more unclear (e. g. chlorpromazine plus haloperidole and the risk for seizures). Against the background of this discussion and the recent discussion if the tranquilizing effect of a drug combination could also be achieved by a higher dose of one drug as monotherapy, the study design with a two- and a three-drug-combination and even the doses of these combinations left at the discretion of the clinician seems questionable.**

The trial did address the adverse events pertaining to adding a third active ingredient to the haloperidol plus promethazine combination. The protocol does indeed state the doses were at the discretion of the clinicians. However, during the trial itself, the doses were fixed at 5mg Haloperidol, 25mg promethazine and 100mg chlorpromazine.

**Concerning protection of data privacy, it would be interesting how the abbreviations for the patients were built up and which data was exchanged via the Whatsapp group.**

The WhatsApp group allowed updates to trial recruitment as well as open questions pertaining to the trial. Patient names were not used nor were they required since trial numbers were allocated. For instance, if a patient was admitted and randomised, the resident in charge would message the group with "New patient number 14, female". At this point, the trial co-ordinator (Myself) would note the date on the group. This was to keep track on trial recruitment.

**(Note to my "partly" answer on the last question: I missed the sheets that had to be filled in by the doctors. I did not understand why the form "Dr. Stopwatch" and "TREC-Lebanon main data collection form" only were present in the HPC- and not in the HP-envelope.)**

We apologise if this was not as clarified as thought. Both the Dr. Stopwatch form and the main data collection form was present in both the HPC and HP envelope.

Once again, we thank you for your time and efforts and hope we have clarified the best we can. We look forward to sharing the trial itself when published.

Regards,

Joseph Dib
